# Testosterone Level Reduction Increases the 10-Year Risk of Cardiovascular Diseases: A Retrospective Cohort Study in a Taiwanese Young Male Population

**DOI:** 10.3389/fcvm.2022.869251

**Published:** 2022-04-14

**Authors:** Han-Hsuan Yang, Shih-Kai Tu, Hsin-Hung Chen, Chia-Lien Hung, Chia-Wen Kuo, Yu-Tse Tsan, Wei-Min Chu, Meng-Chih Lee, Chun-Cheng Liao

**Affiliations:** ^1^Department of Family Medicine, Taichung Armed Forces General Hospital, Taichung City, Taiwan; ^2^Division of Occupational Medicine, Department of Emergency Medicine, Taichung Veterans General Hospital, Taichung City, Taiwan; ^3^School of Professional Education and Continuing Studies, National Taiwan University, Taipei City, Taiwan; ^4^Department of Medicine, National Defense Medical Center, Taipei City, Taiwan; ^5^Division of Endocrinology and Metabolism, Department of Internal Medicine, Asia University Hospital, Taichung City, Taiwan; ^6^School of Medicine, Chung Shan Medical University, Taichung City, Taiwan; ^7^Chung Sheng Clinic, Nantou City, Taiwan; ^8^Department of Medical Education and Research, Taichung Armed Forces General Hospital, Taichung City, Taiwan; ^9^Division of Nephrology, Department of Internal Medicine, Taichung Armed Forces General Hospital, Taichung City, Taiwan; ^10^Department of Family Medicine, Taichung Veterans General Hospital, Taichung, Taiwan; ^11^School of Medicine, National Yang Ming Chiao Tung University, Taipei City, Taiwan; ^12^Institute of Health Policy and Management, National Taiwan University, Taipei City, Taiwan; ^13^Department of Family Medicine, Taichung Hospital, Ministry of Health and Welfare, Taichung City, Taiwan; ^14^Institute of Population Health Sciences, National Health Research Institutes, Miaoli County, Taiwan; ^15^College of Management, Chaoyang University of Technology, Taichung City, Taiwan

**Keywords:** testosterone, cohort study, cardiovascular disease, cardiovascular disease risk, Framingham Risk Score, ASCVD Risk Estimator

## Abstract

Low testosterone levels are associated with increased risk of cardiovascular disease; however, most previous studies assessed the relationship of testosterone levels with a history of cardiovascular (CV) events rather than with CV risk prediction scores consequently neglecting the effect of testosterone on CV risk in healthy young individuals. The aim of this study was to investigate the relationship between testosterone levels and predict the 10-year risk of cardiovascular disease. This retrospective cohort study was conducted through a large medical health examination system in four metropolises in Taiwan. Two risk scores were used to predict the 10-year cardiovascular risk of participants: the Framingham Risk Score (FRS) (2008) and the Atherosclerotic Cardiovascular Disease (ASCVD) Risk Estimator (2013). Multivariate-adjusted logistic regression was used to calculate odds ratios (ORs) for the correlation of testosterone level reduction with the increase in predicted CV risk. We used the MJ Health Research Foundation database to collect reports of 125,414 individuals who underwent medical checkups between 2007 and 2016. The final sample size included 1,253 male participants. A reduction in testosterone level between two subsequent medical checkups was associated with higher CV risk estimated by the FRS and ASCVD Risk Estimator in young participants aged 30–49 years (OR = 0.804, 95% CI: 0.711–0.909, p < 0.01 and OR = 0.841, 95% CI: 0.742–0.953, p < 0.01, respectively). Reduction in total testosterone levels increases CV risk in men aged 30 to 49 years, while the CV risk is not influenced by low testosterone levels at baseline.

## Introduction

Testosterone is one of the most important hormones in the human body. It is not only a key hormone involved in the male and female reproductive system, but its serum levels have also implications for chronic diseases and disorders such as diabetes, hypertension, hyperlipidemia, chronic kidney disease, cancer, and metabolic syndrome (1–4). In recent years, some studies have indicated that low testosterone levels might increase the risk of coronary artery disease, death from congestive heart failure, and all-cause mortality (5, 6).

Cardiovascular disease (CVD) remains one of the most common causes of death worldwide (7). Numerous tools have been created to predict cardiovascular (CV) risk, making it possible to determine an individual's chances of developing CVD and to classify people into different risk categories regardless of their current health status. Since the introduction of the original Framingham Risk Score (FRS) in 1998, numerous other calculators have been designed for different regions or races and using different criteria, such as the current version of the FRS (2008), Atherosclerotic Cardiovascular Disease (ASCVD) Risk Estimator from the American College of Cardiology (ACC) and American Heart Association (AHA) (2013), JBS3 risk calculator (2014), MESA risk score (2015), and China-PAR algorithm (Prediction for ASCVD Risk in China) (2016) (8).

To date, no studies have assessed which calculator is most suitable for the Taiwanese population. Moreover, most previous studies focused on the association between testosterone levels and a history of CV events rather than CV risk prediction scores. It is also known that although low serum testosterone levels may indicate poor general health (9), testosterone is not measured during routine medical checkups. As a result, little is known on the significance of testosterone and its possible effect on CV risk in healthy young men. Therefore, we aimed to investigate the relationship between testosterone levels and the 10-year risk of CVD estimated by the Framingham Risk Score and the ASCVD Risk Estimator from the ACC/AHA in this population.

## Materials and Methods

### Database

In this retrospective cohort study, we obtained data from the database of the MJ Health Research Foundation, which is a large private membership medical clinic in Taiwan (Republic of China). The database covers four health screening centers (in Taipei, Taoyuan, Taichung, and Kaohsiung), where members can receive regular medical examinations based on their preference. Participants underwent medical checkups on own will from January 2007 to December 2016. The age of participants at the first examination was 30 −79 years. The exclusion criteria were as follows: female sex, age younger than 30 years at the time of the first examination or older than 79 years at the time of the second examination, lack of data required for risk score calculation, such as age, sex, race, history of diabetes mellitus, hypertension and the use of antihypertensive drugs, blood pressure, total testosterone, triglyceride, high-density lipoprotein cholesterol (HDL-C), low-density lipoprotein cholesterol (LDL-C), and glucose levels, body mass index (BMI), and smoking status. Personal data of participants in this study were anonymous. All participants in this cohort had provided consent by signing consent forms to authorize the data analysis before the health examination. All data used in this study were authorized by the Taichung Armed Forces General Hospital (Taichung, Taiwan) and obtained from the MJ Health Research Foundation (authorization code: MJHRF2019016A) (10). This study was approved by the Tri-Service General Hospital Institutional Review Board (TSGHIRB number: A202005160) and the procedures were performed according to the principles stated in the Declaration of Helsinki.

### Data Collection

All participants underwent physical examination, body measurements, and laboratory tests of blood samples under fasting state. They also answered a detailed questionnaire about their medical history, including hormone therapy. Systolic and diastolic blood pressure was measured after 10-min rest. The BMI was calculated as weight in kilograms divided by square of height in meters. Total testosterone was expressed as ng/mL; triglycerides, glucose, uric acid, as well as high-density lipoprotein cholesterol and LDL-C, as mg/dL; estimated glomerular filtration rate (eGFR), as mL/min/1.732 m^2^; and prostate-specific antigen, as μg/L. A reduction in testosterone level indicated that the serum level of total testosterone at the second medical checkup was lower than that at the first medical checkup. Data on medical history such as the use of antihypertensive and oral hypoglycemic drugs, family history of chronic diseases, and smoking status (current and former smoking vs. nonsmoking) was listed as one of the data obtained via the questionnaire completed by participants at the time of the examination. Data standardization was achieved by the use of identical screening procedures with the same model of instruments, as well as formal training of health practitioners at all clinics.

### Cardiovascular Risk Assessment

In this study, two risk scores were used to predict the 10-year CV risk of participants: the FRS (2008) (11) and ASCVD Risk Estimator from the ACC/AHA (2013) (12). The formulas of both calculators reference from website of UpToDate online (13), for the FRS and (14) for the ASCVD Risk Estimator. Both risk scores include age, sex, systolic blood pressure, total cholesterol, high-density lipoprotein cholesterol, use of antihypertensive drugs, smoking, and presence of diabetes, while the ASCVD Risk Estimator additionally includes race. We chose to compare these two risk scores because they are widely used tools that were published at a similar time. Moreover, all data required for the calculation of the risk scores were available in our database, and all the tests and examinations required to obtain data for the risk score calculation were cost effective and easily accessible.

### Classification of Cardiovascular Risk

Both risk scores can predict the 10-year CVD risk, which are normally presented as percentage. To apply this to clinical use, we divided the estimated CV risk into four different risk categories according to previous studies. For the FRS, the results were classified as very low risk (≤1%), low risk (>1% to <10%), intermediate risk (10% to <20%), and high risk (≥20%) (15, 16). For the ASCVD Risk Estimator, the categories were as follows: low risk (<5%), borderline risk (≥5% to <7.5%), intermediate risk (≥7.5% to <20%), and high risk (≥20%) (17).

### Outcomes

In this study, we designed two types of outcomes. The first one is to assess whether overall CV risk increases at the second medical checkup or not. If the result of CV risk estimated at the second time is higher than the first medical checkup, the participants would be classified into “increased group” (difference > 0%), and the others would be classified into “control group” (difference ≤ 0).

The second outcome is to assess whether CV risk categories increase at the second medical checkup or not. If the CV risk category at the second medical checkup is higher than the first time, the participants would be classified into the “increased group”, and the others would be classified into “control group”. For example, one participant was assessed as low CV risk category at the first medical checkup, but the category elevated to intermediate risk at the second medical checkup, he or she would be classified into “increased group”, if the participant is assessed as low CV risk category at the second time, he or she would be classified into “control group”.

### Statistical Analysis

Data from two subsequent medical checkups were collected, and 10-year CV risk was estimated by the FRS and ASCVD Risk estimator. The results between risk scores were compared using the Pearson χ^2^ test. Categorical variables were analyzed using the McNemar test.

Men who were free of CV disease at age of 50 have remarkably longer survival and very low remaining risk for CV disease (18), and people aged older than 65 were defined as the elderly by World Health Organization (WHO). Thus, we divided participants into three groups by age, including age 30–49, age 50–64, and age ≥65.

A logistic regression analysis was performed to assess a correlation between the increase in CV risk estimated by the FRS and the reduction in total testosterone. The reduction in total testosterone indicated that the concentration of serum total testosterone at the second checkup was lower than at the first checkup. The definition of outcomes we used in logistic regression are mentioned in “Outcome” section. Some factors had already been included in FRS and ASCVD Risk Estimator, including age, sex, habit of smoking, total cholesterol, high-density lipoprotein cholesterol, blood pressure, and diagnosis of diabetes. In logistic regression analysis, we adjusted BMI, LDL-C, eGFR, and uric acid as confounders, for both of them are not included in CV prediction tools yet are commonly considered to be related to CV risk. Considering the influence of interval of medical checkups (follow year), we also adjusted it as a confounder in the logistic regression analysis (**Table 2**).

In addition, we also analyzed young participants who aged ≤ 49 based on different follow years. According to the interval of twice medical checkups, participants were divided into three groups, including one-year apart, two-year apart, and 3 and more years apart. Logistic regression analysis adjusted to BMI, LDL-C, eGFR, and uric acid were also performed (**Table 3**). A *p* < 0.05 was considered significant. All analyses were conducted at the Department of Medical Education and Research, Taichung Armed Forces General Hospital.

## Results

### Demographic and Clinical Characteristics of the Study Group

**Figure 1:**
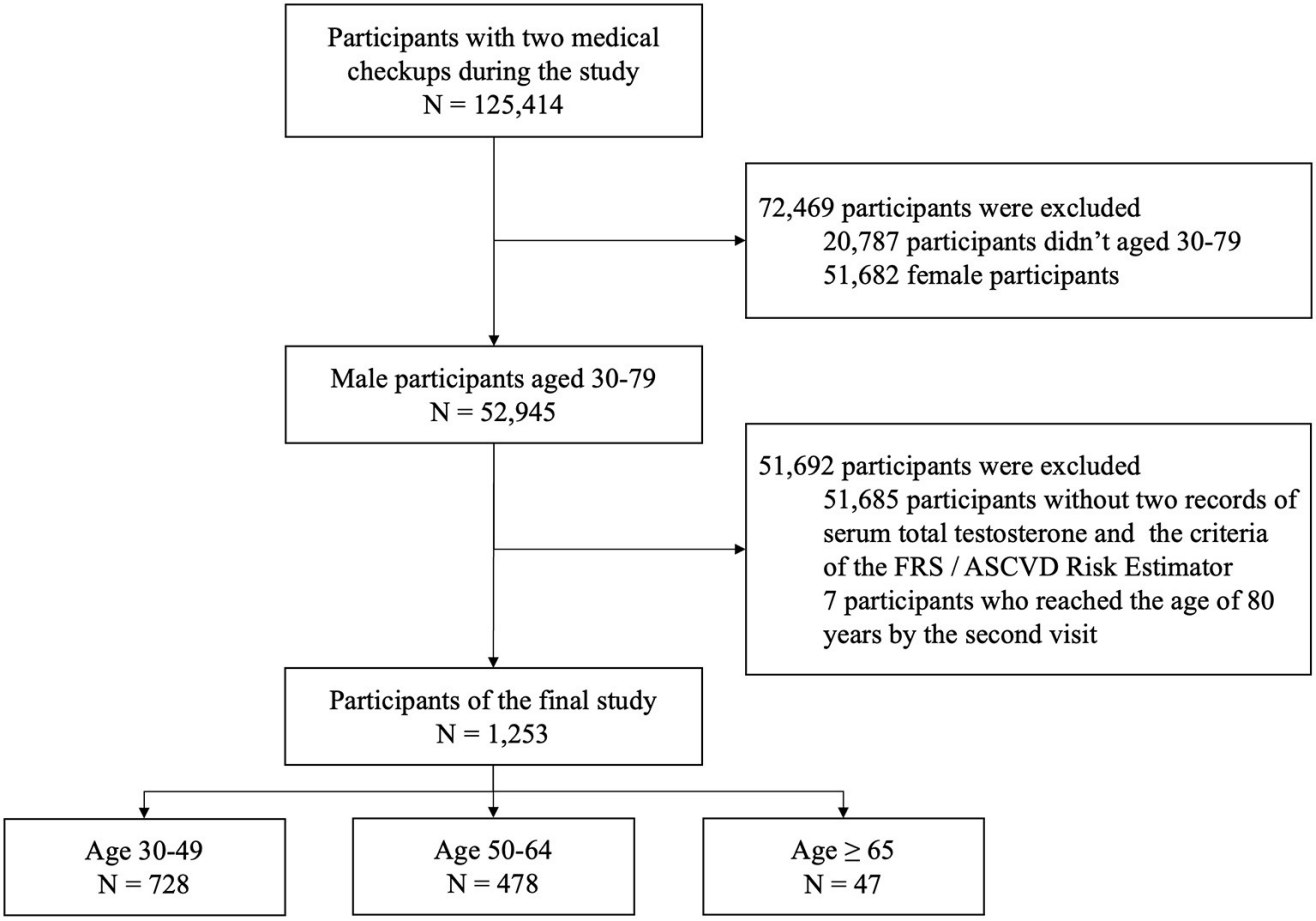
The flow chart illustrates the number of included and excluded participants, the reasons for exclusion, and the final number of participants. FRS, Framingham Risk Score (2008); ASCVD Risk Estimator, Atherosclerotic cardiovascular disease Risk Estimator (2013).

**Figure 2:**
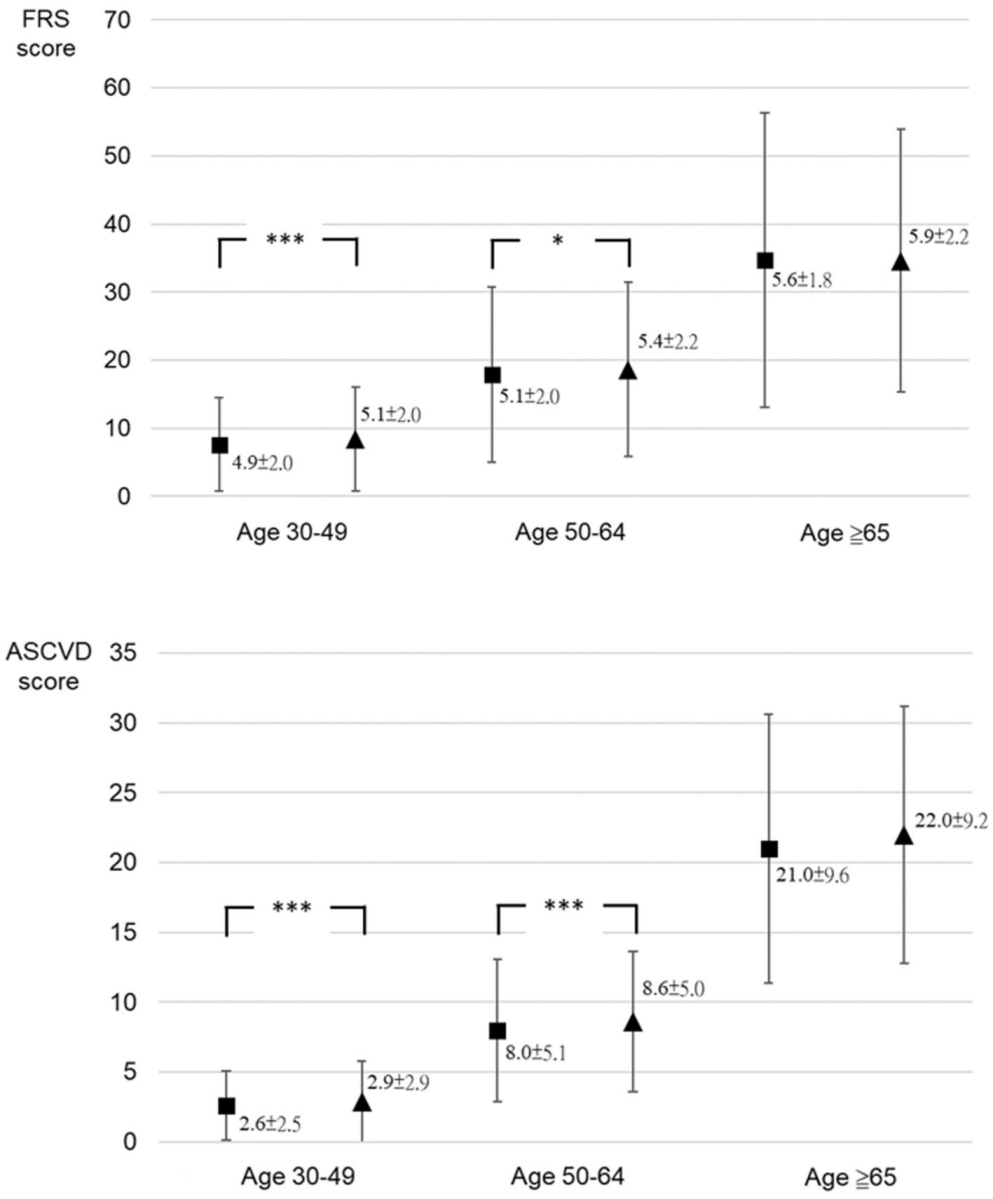
The comparison between cardiovascular risk score of the Framingham Risk Score and ASCVD Risk Estimator at the first and second medical checkups by age group. Framingham Risk Score (FRS) score: cardiovascular risk score (%) calculated using the FRS (2008); Atherosclerotic cardiovascular disease (ASCVD) score: cardiovascular risk score (%) calculated using ASCVD Risk Estimator (2013). ***p < 0.001; *p < 0.05.

A total of 125,414 participants underwent medical checkup at the MJ Health Screening Center during the study, including 52,945 male participants aged 30–79 years. However, only 1,260 participants who met the criteria of the FRS and ASCVD Risk Estimator and had their serum total testosterone tested were included in this cohort study. After the exclusion of seven participants who reached the age of 80 years by the second visit, the final study sample included 1,253 male participants (Figure 1). There was no evidence indicating that any of the final participants received hormone therapy. Age, testosterone levels, blood pressure, and other clinical and demographic characteristics of participants are presented. The mean age of participants was 47.3 (SD = 9.5) years, and the mean time between the checkups was 1.5 (SD = 0.9) years. Of this cohort, 728 participants were younger than 49 years; 478 were between 50 and 64 years, and 47 were 65 years or older at the first medical checkup (Table 1). Diastolic blood pressure differed significantly between visits in participants aged 30 to 49 years, but no differences were noted in the remaining age groups. Serum total testosterone and prostate-specific antigen levels were significantly higher at the second visit in participants aged 30–49 years and 50–64 years, but no differences between visits were noted among participants aged 65 years or older. Cardiovascular risk assessed by both calculators was higher at the second visit in participants younger than 65 years (*p* < 0.05), but no differences were noted in older individuals (*p* > 0.05) (Figure 2). A detailed comparison of study group characteristics according to age and medical checkup is presented in Table 1.

**Table 1:** Comparison of the clinical characteristics of the study group according to age.

**Parameter**	**Age 30–49 (*****n*** **=** **728)**			**Age 50–64 (*****n*** **=** **478)**			**Age** **≥65 (*****n*** **=** **47)**		
	**1st checkup**	**2nd checkup**	**p**	**1st checkup**	**2nd checkup**	**P**	**1st checkup**	**2nd checkup**	**p**
Time between checkups, y		1.5 (0.9)			1.5 (0.9)			1.5 (0.9)	
BMI, kg/m^2^	25.1 (3.8)	25.2 (3.8)	0.414	24.5 (2.9)	24.5 (2.9)	0.076	24.1 (2.5)	23.9 (2.4)	0.192
SBP, mmHg	120.1 (15.1)	120.0 (16.3)	0.820	122.0 (15.6)	121.3 (15.1)	0.206	131.9 (20.2)	131.2 (19.6)	0.753
DBP, mmHg	77.6 (10.8)	78.9 (10.9)	<0.001^***^	79.7 (10.2)	79.4 (10.0)	0.460	79.3 (10.6)	79.5 (9.3)	0.924
HDL-C, mg/dL	50.5 (10.8)	50.9 (10.7)	0.082	52.5 (11.6)	53.0 (12.0)	0.103	54.3 (11.5)	55.2 (11.5)	0.350
LDL-C, mg/dL	124.9 (31.2)	124.2 (31.9)	0.421	122.8 (32.6)	120.2 (31.9)	0.021^*^	126.7 (35.9)	118.1 (34.4)	0.019^*^
TG, mg/dL	146.9 (103.2)	146.0 (114.2)	0.803	138.8 (116.6)	127.0 (73.6)	0.016^*^	110.7 (46.9)	105.4 (47.5)	0.387
FBS, mg/dL	103.9 (18.7)	104.6 (21.8)	0.157	109.5 (20.8)	109.6 (19.2)	0.953	111.6 (17.4)	112.5 (19.1)	0.621
eGFR, mL/min/1.732m^2^	81.6 (11.5)	81.0 (11.2)	0.064	75.6 (10.3)	75.5 (11.6)	0.693	70.8 (13.7)	70.7 (13.7)	0.943
Uric acid, mg/dL	6.7 (1.4)	6.7 (1.4)	0.796	6.4 (1.3)	6.4 (1.3)	0.149	6.4 (1.3)	6.4 (1.4)	0.926
Testosterone, ng/mL	4.9 (2.0)	5.1 (2.0)	<0.001^***^	5.1 (2.0)	5.4 (2.2)	<0.001^***^	5.6 (1.8)	5.9 (2.2)	0.129
PSA, μg/L	0.9 (0.6)	1.0 (0.8)	0.002^**^	1.4 (1.6)	1.5 (1.7)	0.011^*^	3.0 (5.1)	2.4 (2.0)	0.344
FRS	7.6 (6.8)	8.4 (7.6)	<0.001^***^	17.9 (12.9)	18.6 (12.7)	0.050^*^	34.7 (21.6)	34.6 (19.3)	0.930
ASCVD Risk Estimator	2.6 (2.5)	2.9 (2.9)	<0.001^***^	8.0 (5.1)	8.6 (5.0)	<0.001^***^	21.0 (9.6)	22.0 (9.2)	0.204

*ASCVD, atherosclerotic cardiovascular disease; BMI, body mass index; SBP, systolic blood pressure; DBP, diastolic blood pressure; FRS, Framingham Risk Score HDL-C, high-density lipoprotein cholesterol; LDL-C, low-density lipoprotein cholesterol; TG, triglyceride; FBS, fasting blood sugar; eGFR, estimated glomerular filtration rate; Testosterone, total testosterone; PSA, prostate-specific antigen*.

*** *p < 0.001*;

** *p < 0.01*;

* *p < 0.05*.

### Changes in Overall Cardiovascular Risk and by Risk Categories

Changes in overall CV risk and by individual risk categories of the FRS and ASCVD Risk Estimator among participants divided into three age groups (30–49 years, 50–64 years, and ≥65 years). The increase in CV risk estimated by two risk scores was consistent in all participants (61.2% by the FRS and 67.6% by ASCVD Risk Estimator) and age groups. About the changes of CV risk categories, 167 (13.3%) of all participants elevated CV risk categories by FRS, and 170 (13.6%) of all participants elevated CV risk categories by ASCVD Risk Estimator at the second medical checkup.

The McNemar test revealed that both the FRS and the ASCVD Risk Estimator predicted categories of CV risk with a similar accuracy of 0.872 (Pearson's correlation r = 0.927, *p* < 0.001). This indicates a significant correlation between the risk scores not only in terms of predicting the overall CV risk but also in terms of individual risk categories.

### Logistic Regression Analysis of Correlations Between the Increase in Cardiovascular Risk Estimated by the Framingham Risk Score and Selected Parameters

In participants aged 30–49 years, a reduction in the total testosterone level(Δ Testosterone) was significantly negatively correlated with the increase in CV risk (OR = 0.804, 95% CI = 0.711–0.909, p < 0.01). A reduction in the total testosterone level elevated the odds of increased estimated CV risk by 19.6%. However, no significant results were noted for participants older than 49 years (Age 50–64, OR = 0.914, 95% CI = 0.806–1.038. Age ≥ 65, OR = 0.987, 95% CI = 0.654–1.490). Moreover, the baseline level of total testosterone was not significantly associated with the overall CV risk in the whole study cohort. In participants aged 30–49 years, BMI at baseline had a significant protective effect on cardiovascular risk (OR = 0.934, 95% CI = 0.892-−0.979, *p* < 0.01) (Table 2). Baseline LDL-C levels also had a protective effect in this age group (OR = 0.988, 95% CI = 0.983–0.993, *p* < 0.001). Both eGFR and uric acid did not have influence on CV risk significantly in this study. In participants aged 30-49, the group of follow year = 2 years and follow year ≥ 3 years showed significantly higher odds of increased CV risk while the group of follow year = 1 year being the reference, which means the interval of medical checkups is one of risk factors of increased CV risk in young male people. (OR = 1.597, 95% CI = 1.113-2.295, p < 0.05; OR = 2.983, 95% CI = 1.556–5.717, *p* < 0.01, respectively) (Table 2). After classification into four risk categories, no significant correlations between CV risk and reductions in the total testosterone level were shown (Age 30-49, OR = 1.039, 95% CI = 0.878-1.230. Age 50–64, OR = 0.861, 95% CI = 0.717–1.033. Age ≥ 65, OR = 1.464, 95% CI = 0.659–3.251. Total participants, OR = 0.958, 95% CI = 0.851–1.077). Detailed data are also shown in Table 2. This indicates that reductions in testosterone levels affect CV risk in the young male population, but without difference in individual risk category between medical checkups with a mean interval of 18 months apart. This might be due to the fact that it takes 2.5–12.5% of elevation in CV risk to increase one grade in CV risk category. The results above revealed that although a reduction of total testosterone level increases CV risk, the impact is not strong enough to cause actual elevation in CV risk category in the short term. Thus, the result of CV risk categories is not significant. However, further studies are needed to confirm this finding.

**Table 2:** Logistic regression analysis of correlations between the increase in cardiovascular risk assessed and selected parameters.

**Risk scores**	**Framingham Risk Score (2008)**		**ASCVD Risk Estimator (2013)**	
	**OR**	**95.0% C.I**.	**OR**	**95.0% C.I**.
**Age 30–49 (*****n*** **=** **728)**				
**Overall CV risk (Increased group vs. control group)**				
Δ Testosterone	0.804^**^	0.711–0.909	0.841^**^	0.742-0.953
Testosterone (baseline)	1.019	0.928–1.119	0.974	0.886-1.071
BMI (baseline)	0.934^**^	0.892–0.979	0.935^**^	0.890-0.979
LDL-C (baseline)	0.988^***^	0.983–0.993	0.985^***^	0.979-0.990
eGFR	0.999	0.985–1.013	0.997	0.983-1.012
Uric acid	1.131^*^	0.999–1.280	1.133	0.997-1.288
**Follow year**				
1 year	Ref.		Ref.	
2 years	1.597^*^	1.113–2.295	1.769^*^	1.211-2.584
≥3 years	2.983^**^	1.556–5.717	3.944^***^	1.871-8.316
**CV risk categories (Increased group vs. control group)**				
Δ Testosterone	1.039	0.878–1.230	0.990	0.810-1.209
Testosterone (baseline)	1.052	0.923–1.199	0.993	0.841-1.172
BMI (baseline)	0.983	0.916–1.055	0.992	0.916-1.074
LDL-C (baseline)	0.998	0.990–1.006	1.003	0.994-1.012
eGFR	0.994	0.973–1.015	0.995	0.972-1.019
Uric acid	1.095	0.916–1.310	1.230^*^	1.004-1.505
**Follow year**				
1 year	Ref.		Ref.	
2 years	1.389	0.830–2.325	1.973^*^	1.083-3.593
≥3 years	2.114^*^	1.046–4.273	3.348^*^	1.556-7.204
**Age 50–64 (*****n*** **=** **478)**				
**Overall CV risk (Increased group vs. control group)**				
Δ Testosterone	0.914	0.806–1.038	0.906	0.794-1.034
Testosterone (baseline)	1.057	0.949–1.176	1.056	0.942-1.185
BMI (baseline)	0.981	0.915–1.053	0.953	0.884-1.026
LDL-C (baseline)	0.998	0.992–1.004	0.994	0.988-1.000
eGFR	1.013	0.994–1.032	1.007	0.987-1.027
Uric acid	0.958	0.821–1.119	0.929	0.788-1.094
**Follow year**				
1 year	Ref.		Ref.	
2 years	1.151	0.727–1.822	1.538	0.936-2.527
≥3 years	1.893^*^	1.055–3.396	2.597^**^	1.335-5.051
**CV risk categories (Increased group vs. control group)**				
Δ Testosterone	0.861	0.717–1.033	0.938	0.805-1.097
Testosterone (baseline)	1.016	0.885–1.166	0.973	0.856-1.107
BMI (baseline)	0.981	0.891–1.079	0.999	0.916-1.089
LDL-C (baseline)	0.999	0.992–1.007	0.999	0.992-1.005
eGFR	0.978	0.953–1.003	0.989	0.967-1.012
Uric acid	0.852	0.692–1.050	0.890	0.737-1.074
**Follow year**				
1 year	Ref.		Ref.	
2 years	1.126	0.616–2.058	1.330	0.766-2.308
≥3 years	1.266	0.622–2.578	2.627^*^	1.447-4.771
**Age** **≥65 (*****n*** **=** **47)**				
**Overall CV risk (Increased group vs. control group)**				
Δ Testosterone	0.987	0.654–1.490	0.864	0.561-1.330
Testosterone (baseline)	1.129	0.716–1.778	1.123	0.705-1.789
BMI (baseline)	1.248	0.943–1.657	1.216	0.904-1.637
LDL-C (baseline)	1.003	0.983–1.024	1.001	0.980-1.023
eGFR	0.991	0.944–1.040	0.999	0.949-1.052
Uric acid	1.284	0.729–2.264	1.208	0.671-2.176
**Follow year**				
1 year	Ref.		Ref.	
2 years	1.358	0.178–10.364	2.592	0.236-28.471
≥3 years	1.383	0.200–9.541	1.960	0.252-15.250
**CV risk categories (Increased group vs. control group)**				
Δ Testosterone	1.464	0.659–3.251	1.410	0.845-2.351
Testosterone (baseline)	0.934	0.415–2.102	0.760	0.381-1.517
BMI (baseline)	1.536	0.940–2.509	0.966	0.670-1.392
LDL-C (baseline)	0.972	0.928–1.019	0.976	0.943-1.011
eGFR	1.027	0.954–1.104	0.992	0.926-1.064
Uric acid	2.349	0.800–6.899	0.923	0.358-2.377
**Follow year**				
1 year	Ref.		Ref.	
2 years	1.075	0.053–21.745	20.534^*^	1.578-267.244
≥3 years	–	–	3.428	0.287-41.001
**Total participants (n** **=** **1,253)**				
**Overall CV risk (Increased group vs. control group)**				
Δ Testosterone	0.859^***^	0.789–0.935	0.873^**^	0.800-0.952
Testosterone (baseline)	1.032	0.964–1.105	1.009	0.940-1.084
BMI (baseline)	0.954^*^	0.919–0.990	0.945^**^	0.909-0.982
LDL-C (baseline)	0.994^**^	0.990–0.997	0.990^***^	0.986-0.994
eGFR	1.008	0.998–1.019	1.003	0.992-1.014
Uric acid	1.077	0.981–1.181	1.062	0.964-1.170
**Follow year**				
1 year	Ref.		Ref.	
2 years	1.441^**^	1.094–1.898	1.696^***^	1.264-2.276
≥3 years	2.300^***^	1.520–3.480	3.131^***^	1.948-5.030
**CV risk categories (Increased group vs. control group)**				
Δ Testosterone	0.958	0.851–1.077	1.004	0.894-1.128
Testosterone (baseline)	1.028	0.937–1.127	0.982	0.892-1.081
BMI (baseline)	0.987	0.934–1.043	0.993	0.939-1.049
LDL-C (baseline)	0.998	0.993–1.003	0.999	0.994-1.004
eGFR	0.985^*^	0.970–1.000	0.979^**^	0.964-0.994
Uric acid	0.983	0.862–1.120	0.976	0.856-1.113
**Follow year**				
1 year	Ref.		Ref.	
2 years	1.232	0.842–1.801	1.514^*^	1.033-2.220
≥3 years	1.541	0.941–2.522	2.899^***^	1.857-4.5261

*CV, cardiovascular; Δ Testosterone, a reduction in total testosterone level between the second and first medical checkup; BMI, body mass index; LDL-C, low-density lipoprotein cholesterol; eGFR, estimated glomerular filtration rate; Ref., reference*.

*** *p < 0.001*;

** *p < 0.01*;

* *p < 0.05*.

### Logistic Regression Analysis of the Correlations Between the Increase in Cardiovascular Risk Estimated by the ASCVD Risk Estimator and Selected Parameters

The logistic regression analysis of correlations between increase in CV risk estimated by the ASCVD Risk Estimator and reductions in total testosterone, baseline testosterone, BMI, and LDL-C provided similar results to those observed for the CV risk assessed by the FRS. The reduction in total testosterone levels was correlated with a significantly higher odds of increased CV risk of participants aged 30–49 years (OR = 0.841, 95% CI = 0.742–0.953, *p* < 0.01) (Table 2). A reduction in the total testosterone level elevated the odds of increased estimated CV risk by 15.9%. No significant correlations were noted for participants older than 49 years (Age 50–64, OR = 0.906, 95% CI = 0.794–1.034. Age ≥ 65, OR = 0.864, 95% CI = 0.561–1.330). In participants aged 30–49, the group of follow year = 2 years and follow year ≥ 3 years showed significantly higher odds of increased CV risk while the group of follow year = 1 year being the reference (OR = 1.769, 95% CI = 1.211–2.584, p < 0.05; OR = 3.944, 95% CI = 1.871–8.316, p <0.001, respectively) (Table 2). After classification into four risk categories, no significant correlations between CV risk and the above dependent variables were shown (Age 30–49, OR = 0.990, 95% CI = 0.810–1.209. Age 50–64, OR = 0.938, 95% CI = 0.805–1.097. Age ≥ 65, OR = 1.410, 95% CI = 0.845–2.351. Total participants, OR = 1.004, 95% CI = 0.894–1.128).

### Logistic Regression Analysis of Correlations Between the Increase in Cardiovascular Risk and Selected Variables Depending on the Time Between Medical Checkups

We independently analyzed correlations between the increase in CV risk and selected variables depending on the time between medical checkups in participants aged 30–49 years. The increase in CV risk calculated by the FRS was significantly associated with the reduction in testosterone levels both in participants who underwent medical checkups one-year apart (OR = 0.893, 95% CI: 0.802–0.995, p < 0.05) and those with checkups 2 years apart (OR = 0.805, 95% CI: 0.680–0.954, *p* < 0.05) (Table 3), but there is no significant association in the group of follow year 3 ≥ years (OR = 0.833, 95% CI = 0.654-1.061). For CV risk estimated by the ASCVD Risk Estimator, only participants who underwent medical checkups 2 years apart showed significant correlations (OR = 0.707, 95% CI: 0.585–0.855, *p* < 0.001) (Table 3).

**Table 3:** Logistic regression analysis of correlations based on time between medical checkups in participants aged 30–49 years.

	**Follow year** **=** **1 year (*****n*** **=** **448)**		**Follow year** **=** **2 years (*****n*** **=** **214)**		**Follow year** **≥3 years (*****n*** **=** **66)**	
	**OR**	**95.0% C.I**.	**OR**	**95.0% C.I**.	**OR**	**95.0% C.I**.
**Over all CV risk by FRS (Increased group vs. control group)**						
Δ Testosterone	0.893^*^	0.802–0.995	0.805^*^	0.680–0.954	0.833	0.654–1.061
Testosterone (baseline)	1.035	0.953–1.124	1.037	0.900–1.194	1.003	0.775–1.297
BMI (baseline)	0.967	0.922–1.014	0.920^*^	0.856–0.989	0.989	0.862–1.136
LDL-C (baseline)	0.995^*^	0.991–1.000	0.990^*^	0.981–0.998	0.990	0.978–1.003
eGFR	1.008	0.995–1.022	1.013	0.993–1.033	0.989	0.954–1.026
Uric acid	1.105	0.985–1.239	0.996	0.831–1.193	1.100	0.772–1.565
**Over all CV risk by ASCVD (Increased group vs. control group)**						
Δ Testosterone	0.957	0.857–1.068	0.707^***^	0.585–0.855	0.777	0.590–1.023
Testosterone (baseline)	1.031	0.948–1.122	0.948	0.813–1.105	0.983	0.723–1.338
BMI (baseline)	0.971	0.925–1.020	0.900^**^	0.833–0.972	0.884	0.760–1.029
LDL-C (baseline)	0.991^***^	0.986–0.995	0.984^**^	0.975–0.993	0.997	0.983–1.011
eGFR	1.001	0.987–1.014	1.007	0.986–1.028	1.007	0.965–1.051
Uric acid	1.119	0.995–1.259	0.966	0.794–1.175	0.843	0.560–1.270

*FRS: cardiovascular risk score calculated by the Framingham Risk Score (2008); Δ Testosterone: a reduction in total testosterone level between the second and first medical checkup; ASCVD: cardiovascular risk score calculated by the ASCVD Risk Estimator (2013); LDL-C: low-density lipoprotein cholesterol; BMI: body mass index; eGFR: estimated glomerular filtration rate*.

*** *p < 0.001*;

** *p < 0.01*;

* *p < 0.05*.

Baseline LDL-C levels were significantly correlated with lower or no change in CV risk both for one-year and two-year interval between checkups irrespective of the risk score used (Table 3).

## Discussion

Our study showed that the reduction in serum total testosterone levels were correlated with the increase in the 10-year risk of CV events estimated both by the FRS and ASCVD Risk Estimator in young male participants aged 30 to 49 years. However, the baseline level of total testosterone did not significantly affect CV risk in this study.

Cardiovascular disease was ranked highest among the top 10 causes of death in low-middle- to high-income countries worldwide, according to the World Health Organization report from 2018 (7). There are numerous calculators used to assess the risk of CV event or ASCVD, but each of them may be applied in different settings. Guerra-Silva et al. (19) indicated that the FRS has an advantage over the ASCVD Risk Estimator in terms of comprehensive criteria. Topel et al. (20) reported that the FRS provided a worse estimate of racial differences in subclinical CVD. Moreover, Wang et al. (21) suggested that the ASCVD Risk Estimator provided a better estimate of CV risk in the population of the Chinese mainland. As our study showed a similar CV risk for the FRS and the ASCVD Risk Estimator, we suggest that both these tools may be equally useful in the Taiwanese population.

Harman et al. (22) reported that the serum total testosterone level decreased with age in the majority of the male population. This is in contrast to our study, in which all participants had higher serum testosterone levels at the second medical checkup, although there was no evidence that they underwent testosterone replacement therapy. The discrepant results might be due to a short period of time between medical checkups (a mean of 1.5 years). Another possible reason is that participants who frequently underwent medical checkups paid more attention to their health. However, the effect of lifestyle factors and socioeconomic status were not assessed in this study.

According to Kloner et al. (23) and Corona et al. (24) low levels of endogenous testosterone were related to atherosclerosis, coronary artery disease, or CV events. Lee et al. (25) indicated a significant relationship between low levels of total testosterone and high FRS scores. Some previous studies reported that low testosterone levels might increase the risk of CV events, but the effect of baseline testosterone levels on CV risk remains controversial. Shores and Matsumoto (26) hypothesized that a low testosterone level is a biomarker of poor health condition. Our results also showed that low testosterone levels did not directly affect the CV risk estimated by both calculators.

Chock et al. (27) reported the result of significant negative correlation between testosterone level and Framingham risk scores, which is different from the result of the elderly group in our study. The database of Chock's study is from Veterans Affairs (VA), and the mean age of all participants is 61.3. In our study, the mean age of all participants is 47.3, and the number of participants aged ≥ 65 is 47 which accounts for 3.75% (47/1,253) of all participants, suggesting this group being underrepresented. It is possible that the changes of testosterone level would affect CV risk both in young male and the elderly.

We studied the relationship between the reduction in testosterone and the increase in CV risk to determine which causes for the increased CV risk are important in young male population, which could help to avoid the legacy effect. The reason why the reduction in testosterone levels affected the increase in CV risk only in men below 50 years of age might be due to the fact that this hormone in younger individuals is heavily dependent on numerous factors, which are probably more likely to increase CV risk than aging itself. Geniole and Carre (28) indicated that testosterone levels fluctuate rapidly depending on current or future social behaviors. Handelsman et al. (29) showed that a gradual decline in testosterone levels could be clearly noted after the age of 35, and a more significant decline occurred after the age of 80. As stated above, testosterone fluctuations in young men are more related to social, work, or sexual activities, while an age-related decline is more prominent in elderly individuals. It is possible that the age-related decline in testosterone levels might not cause a significant elevation of CV risk, but further studies are needed to confirm this hypothesis. The reduction of testosterone level would not change the variables in the FRS and ASCVD Risk Estimator such as sex, race, the rate of aging, habit of smoking, and diagnosis of diabetes. Thus, we suggest that the reduction of serum testosterone increases estimated CV risk through the changes of blood pressure or lipid profiles including total cholesterol and HDL-C.

We also assessed the increase in CV risk in individuals aged 30–49 years with different time periods between checkups. A reduction in total testosterone significantly increases CV risk after a mean period of 12 months between checkups when assessed by the FRS alone, while a significant result after a mean period of 18 months was noted for the risk assessed both by the FRS and ASCVD Risk Estimator. Based on these results, we suggest that testosterone influences CV risk in young men aged 30–49 years, and the FRS is more sensitive than ASCVD Risk Estimator in the short-term interval. Inconsistent results were shown in different follow-up periods in Table 3, at least two reasons could lead to it in the authors' perspective. First, the number of participants followed at 3 and more years apart is underrepresented which accounts for only 5.27% (66/1,253) of all participants. The second possible reason is that the other factors such as hyperlipidemia and smoking status could have stronger influence on CV risk than testosterone three and more years apart, thus further studies with long term follow-up are needed.

In our study, baseline LDL-C levels were associated with a lower odds of increase in CV risk among young participants aged 30–49 years. A previous cohort study reported that baseline LDL-C levels did not affect CV risk among patients with hypertension during the 3-year follow-up (30). Moreover, Nanna et al. (31) revealed that LDL-C levels were not associated with a 5-year risk of ASCVD in participants aged 75 years or older. In our study, baseline BMI also associated with a lower CV risk. Peterson et al. (32) indicated BMI to be a modifiable factor in cardiac structure and function, but this was in contrast to another study that reported an association between low BMI and high risk of CVD in Africans (33). The association between baseline BMI, LDL-C and CV risk remain controversial. Thus, most CV risk estimators do not include BMI and LDL-C. In our study, LDL-C levels and BMI did not differ significantly between two medical checkups. Therefore, we consider that the weakly positive coefficient of baseline LDL-C and BMI are not meaningful in this cohort.

Our study has three limitations. Firstly, elderly individuals constituted only 3.75% of the study group, which is not consistent with the annual report of the Ministry of the Interior in Taiwan from 2016 (34), which showed that individuals aged 65 years or older accounted for 13.20% of the total population. Therefore, the results for CV risk in the whole study group as well as elderly participants might have been biased. Secondly, participants decided on the time and frequency of medical checkups themselves. Therefore, it is possible that our participants had better socioeconomic status and awareness of health care, including prevention of CVD, and thus the results may not be representative of the whole Taiwanese population. Further studies are needed to determine the benefits of routine testosterone measurements in elderly men as well as other individuals who were not represented by this cohort. Finally, participants in this cohort underwent medical checkups 1.5 years apart in average; therefore, more studies with longer interval between checkups are required to confirm the relationship between CV risk and the level of testosterone.

## Conclusions

The concentration of serum testosterone has been known to be correlate to CV disease, yet what we didn't understand was the relationship between the testosterone level and risk of CV disease among the young age group. Fortunately, we could now predict the risk of cardiovascular disease in people who aged above 30 years old by using mature CV disease risk scores such as Framingham Risk Score (FRS) and ASCVD Risk Estimator.

The major finding of this study reveals that a reduction in serum total testosterone levels was associated with a higher 10-year risk of CV events in young men aged 30–49 years, but the risk is not affected by low testosterone levels at baseline. It could be valuable to investigate how testosterone affects the CV risk in young men in further studies.

## Data Availability Statement

The original contributions presented in the study are included in the article/supplementary materials, further inquiries can be directed to the corresponding authors.

## Ethics Statement

This study was approved by the Tri-Service General Hospital Institutional Review Board (TSGHIRB number: A202005160) and the procedures were performed according to the principles stated in the Declaration of Helsinki.

## Author Contributions

H-HY, S-KT, M-CL, and C-CL: conceptualization, writing–review and editing, and project administration. H-HY, C-LH, Y-TT, W-MC, and C-CL: methodology. C-LH and C-WK: software. C-LH, H-HC, and C-CL: validation. H-HY, C-LH, and C-CL: formal analysis. H-HY, H-HC, Y-TT, and W-MC: investigation. S-KT, C-WK, M-CL, and C-CL: resources. H-HY, C-WK, and C-CL: data curation. H-HY and C-CL: writing–original draft preparation. H-HY, H-HC, and C-LH: visualization. Y-TT, W-MC, M-CL, and C-CL: supervision. All authors have read and agreed to the published version of the manuscript.

## Conflict of Interest

The authors declare that the research was conducted in the absence of any commercial or financial relationships that could be construed as a potential conflict of interest.

## Publisher's Note

All claims expressed in this article are solely those of the authors and do not necessarily represent those of their affiliated organizations, or those of the publisher, the editors and the reviewers. Any product that may be evaluated in this article, or claim that may be made by its manufacturer, is not guaranteed or endorsed by the publisher.
